# Case Report: When infection mimics autonomic failure: postural hypotension secondary to skull base osteomyelitis

**DOI:** 10.3389/fmed.2026.1795309

**Published:** 2026-05-19

**Authors:** Avik Roy, Ayuni Zahirah Zahar, Zin Lin Tun, Saikat Mandal, Arkadeep Dhali

**Affiliations:** 1 Department of Medicine for the Elderly, Hull University Teaching Hospitals NHS Trust, Hull, United Kingdom; 2 School of Medicine, University of Nottingham, Nottingham, United Kingdom; 3 NIHR Nottingham Biomedical Research Centre, Nottingham University Hospitals NHS Trust and the University of Nottingham, Nottingham, United Kingdom; 4 Sheffield Teaching Hospitals NHS Foundation Trust, Sheffield, United Kingdom; 5 Faculty of Health, Imperial College London, London, United Kingdom

**Keywords:** autonomic failure, necrotising otitis externa, postural hypotension, recurrent falls, skull base osteomyelitis

## Abstract

**Background:**

Skull base osteomyelitis (SBO) is an uncommon but life-threatening complication of necrotising otitis externa, typically affecting older adults with diabetes. Presentation is often non-specific, and diagnosis is frequently delayed.

**Case:**

An 84-year-old man with type 1 diabetes, vascular comorbidity and advanced frailty presented with a fall preceded by dizziness on standing. He reported a 6-month history of recurrent falls and severe postural light-headedness; persistent right-sided otalgia and offensive otorrhoea had been present for the preceding 3 months, indicating that the ear symptoms followed the orthostatic symptoms. On admission, he had marked postural hypotension despite withdrawal of potentially contributory medication and initiation of fludrocortisone. Initial CT brain imaging was normal, and the sepsis screen was inconclusive. Ten days after admission, he developed a new right-sided facial palsy. Repeat CT head was again unremarkable. Shortly afterwards, he had profuse bleeding from the right ear. Otoscopy, performed for the first time in hospital by an otorhinolaryngology specialist, showed florid otitis externa with a polypoid lesion in the external auditory canal. MRI with skull base sequences revealed extensive necrotising otitis externa with right skull base osteomyelitis extending towards the carotid canal, while CT venography later showed adjacent inflammatory change around the petrous internal carotid artery. Ear swab culture grew *Pseudomonas aeruginosa* sensitive to gentamicin and intermediately sensitive to ceftazidime. High-dose intravenous ceftazidime (2 g three times daily) was started shortly after diagnostic MRI, microbiological sampling, and infectious diseases review, approximately 10 days after admission, and was continued for approximately 7 weeks. Inflammatory markers normalised, and repeat swab cultures were negative. However, follow-up MRI demonstrated partial resolution of the primary osteomyelitis focus with extension into the temporomandibular fossa. Severe postural hypotension persisted despite withdrawal of potentially contributory medication, together with non-pharmacological measures and fludrocortisone. The patient became increasingly deconditioned and bed-bound, developed prolonged delirium and stage 3 acute kidney injury, and died in hospital.

**Conclusion:**

This case highlights skull base osteomyelitis as an important differential diagnosis in older, frail patients with diabetes who present with chronic ear disease, cranial neuropathy and otherwise unexplained refractory orthostatic hypotension. In this patient, inflammation adjacent to the carotid canal/carotid sinus region may have contributed, although causality could not be proven, and alternative contributors to autonomic dysfunction remained possible.

## Highlights

- Persistent otalgia and otorrhoea in older adults with diabetes, particularly when refractory to topical therapy, should prompt early otorhinolaryngology referral and consideration of necrotising otitis externa and skull base osteomyelitis.
- New cranial neuropathy or otherwise unexplained refractory orthostatic hypotension in the context of chronic ear disease should prompt skull base imaging even when routine CT brain imaging is normal.
- Follow-up MRI findings should be interpreted alongside the clinical course and inflammatory markers; persistent or apparently progressive abnormal signal does not always equate to uncontrolled infection.
- Management usually requires prolonged culture-directed therapy, careful multidisciplinary follow-up and selective use of surgery or adjunctive metabolic imaging in refractory or diagnostically discordant cases.

## Background

Skull base osteomyelitis (SBO) is an uncommon but severe infection, most frequently encountered in older adults with diabetes and usually arising as a complication of otogenic disease, classically necrotising (malignant) otitis externa or chronic suppurative otitis media, or, less often, paranasal sinus pathology (1, 2). Early manifestations such as persistent otalgia, otorrhoea and headache are non-specific, and routine CT brain imaging may be normal in the initial stages. These features contribute to diagnostic delay and to the persistently high mortality associated with SBO despite advances in cross-sectional imaging and antimicrobial therapy. Management is challenging and typically requires a multidisciplinary approach that combines careful clinical assessment, high-resolution imaging of the temporal bone and skull base, exclusion of malignancy on biopsy, and prolonged courses of targeted antimicrobial treatment guided by microbiological culture (3). *Pseudomonas aeruginosa* is the predominant pathogen, with *Staphylococcus aureus* the next most frequently isolated organism (2, 4).

Cranial neuropathies, particularly involving the facial nerve, develop when infection extends through the foramina of the skull base to involve contiguous neurovascular structures. Vascular involvement of the petrous or cavernous segment of the internal carotid artery, and occasionally the carotid sinus region, has been described. The carotid sinus is a baroreceptor-rich region whose afferent signalling travels predominantly via Hering’s nerve, a branch of the glossopharyngeal nerve, to the nucleus tractus solitarius; inflammatory extension adjacent to this region could therefore plausibly disturb baroreflex function and contribute to orthostatic hypotension. However, direct demonstration of such involvement is difficult. To our knowledge, only a few prior reports have linked SBO to severe postural hypotension with recurrent syncope attributed to carotid sinus involvement, and those reports did not provide formal autonomic testing or direct radiological confirmation of carotid sinus involvement, so causality remained inferential (4).

We report a case of *Pseudomonas* skull base osteomyelitis in an octogenarian with diabetes and advanced frailty, presenting with recurrent falls and profound postural hypotension that was refractory to standard conservative and pharmacological measures.

## Case presentation

An 84-year-old man was admitted after being found on the floor by carers following an unwitnessed fall. He described sudden dizziness on standing immediately prior to the event and a 6-month history of similar episodes, often leading to falls.

Comorbidities included type 1 diabetes mellitus, ischaemic heart disease, atrial fibrillation, hypertension, a prior ischaemic stroke (with residual dysphasia only), and a long-term indwelling catheter for benign prostatic hyperplasia. His Clinical Frailty Scale score was 7. Home medications comprised cardiovascular or vasoactive agents (isosorbide mononitrate, transdermal and sublingual glyceryl trinitrate, lisinopril, clopidogrel and atorvastatin), neuro-otological and psychoactive agents (betahistine, nortriptyline and sertraline), metabolic therapy (metformin and biphasic insulin), and other long-term medications (ferrous fumarate, lansoprazole, mebeverine and finasteride).

For 3 months, he had experienced right ear pain and malodorous discharge. Primary care records showed multiple courses of oral antibiotics and steroid/antibiotic ear drops for presumed otitis externa; no referral to an otorhinolaryngology specialist was made.

On admission, he was confused but afebrile and haemodynamically stable when supine. Neurological examination was non-focal; there was no cranial nerve palsy. Marked symptomatic orthostatic hypotension was demonstrated at the bedside, with reproduction of his dizziness, although the exact paired supine and standing blood pressure and heart rate values were not captured in the contemporaneous case summary available for this report. The initial CT head was normal. Blood cultures and urine culture were negative, and no alternative septic focus was identified. Formal autonomic testing, including tilt-table testing, was not performed during this admission.

Lisinopril, isosorbide mononitrate, transdermal glyceryl trinitrate and nortriptyline were discontinued. Non-pharmacological measures for postural hypotension were instituted, and fludrocortisone was commenced. Despite this, he remained significantly symptomatic and became progressively bedbound.

Ten days after admission, he developed a new right-sided facial palsy. Considering the risk of a new stroke, repeat CT head imaging was performed, which showed no acute intracranial pathology. Shortly afterwards, he had a large spontaneous bleed from the right external auditory canal. After immediate resuscitation and clinical stabilisation, review by an otorhinolaryngology specialist with otoscopy revealed florid otitis externa and a polypoid mass in the right external auditory canal. This prompted further imaging.

### Investigations

**Figure 1:**
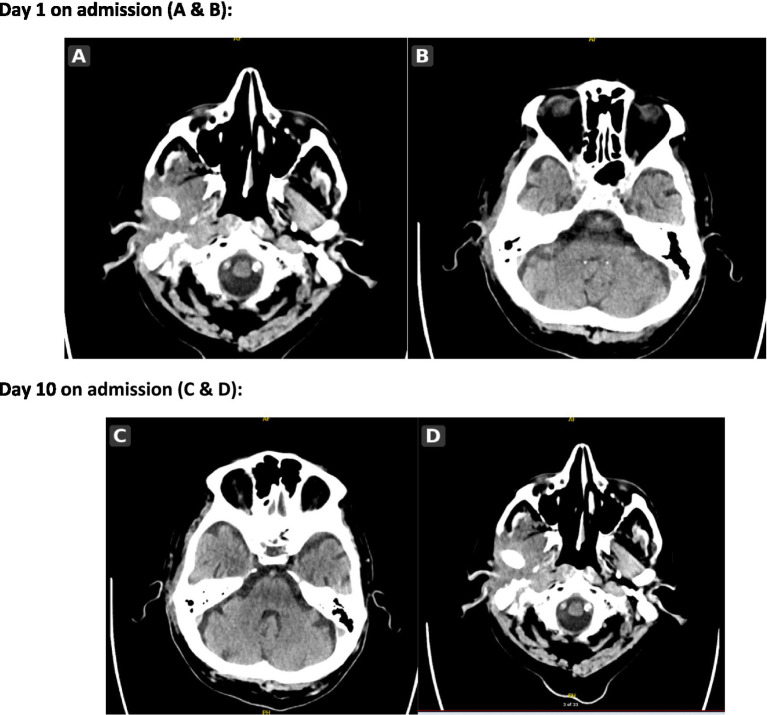
CT head imaging on day 1 **(A,B)** and day 10 **(C,D)** of admission. Initial non-contrast CT head obtained on admission (panels **A,B**) demonstrated no acute intracranial pathology. A repeat CT head obtained 10 days after admission (panels **C,D**), following the development of right-sided facial palsy, again showed no acute intracranial findings. Across both time points, conventional CT did not demonstrate definite skull base abnormality, illustrating its limited sensitivity for early necrotising otitis externa and skull base osteomyelitis in this case.

**Figure 2:**
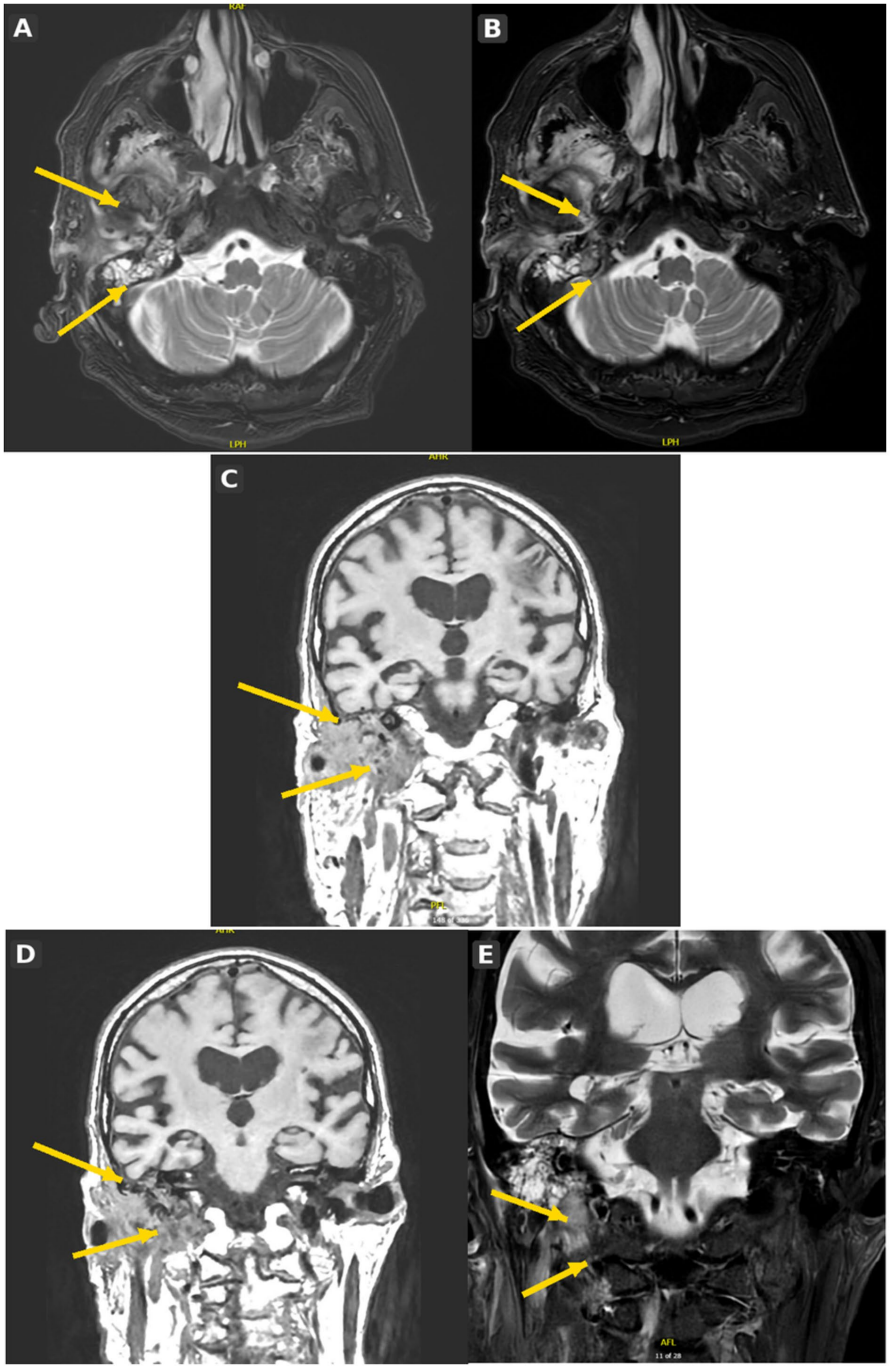
MRI with temporal bone and skull base sequences (**A–E** from top left to bottom right; arrows indicate right-sided abnormal soft-tissue and skull base signal). MRI head with dedicated temporal bone and skull base imaging (panels **A–E**) demonstrated extensive right-sided otogenic skull base infection. Axial T2-weighted FLAIR (panels **A,B**) and post-contrast T1-weighted fat-suppressed sequences (panels **C–E**) showed abnormal signal and enhancement within the right external auditory canal and adjacent skull base soft tissues, with extension towards the carotid canal. CT venographic correlation suggested inflammatory change adjacent to, rather than definite invasion of, the petrous internal carotid artery. The appearances were consistent with necrotising otitis externa complicated by skull base osteomyelitis. MRI provided superior soft-tissue delineation compared with CT for defining disease extent.

MRI head with temporal bone and skull base sequences: extensive necrotising otitis externa with right skull base osteomyelitis centred on the temporal bone and adjacent skull base soft tissues, with inflammatory change extending towards the carotid canal. CT venogram showed no venous sinus thrombosis or major arterial occlusion; appearances were consistent with inflammatory soft-tissue change adjacent to the petrous segment of the internal carotid artery. Direct carotid sheath involvement was not demonstrated radiologically. Microbiology: right ear swab grew *Pseudomonas aeruginosa* sensitive to gentamicin and intermediately sensitive to ceftazidime; repeat swab after systemic therapy showed no growth. Blood and urine cultures remained negative. Specific numerical WBC, ESR and CRP values were not available in the source case summary used for this report, but inflammatory markers were monitored serially and later normalised with treatment (see Figures 1, 2).

### Treatment and outcome

The working diagnosis was pseudomonal skull base osteomyelitis complicating necrotising otitis externa, with facial nerve involvement and possible autonomic dysfunction related to inflammation adjacent to the carotid canal/carotid sinus region.

High-dose intravenous ceftazidime 2 g three times daily was initiated shortly after otorhinolaryngology review, diagnostic MRI and microbiological sampling, following discussion with infectious diseases specialists, and continued for about 7 weeks. Given the culture-confirmed Pseudomonas isolate and specialist recommendation, a high-dose targeted antipseudomonal regimen was pursued, with close monitoring of renal function and inflammatory markers. Supportive care included analgesia, nutritional support, physiotherapy and comprehensive delirium management.

Cessation of vasoactive drugs, fludrocortisone therapy and non-pharmacological measures failed to improve orthostatic hypotension, which remained profound. Inflammatory markers normalised, and ear cultures became negative, but a follow-up MRI after 7–8 weeks showed partial resolution of the primary osteomyelitis focus with extension into the temporomandibular fossa. This discordant picture was interpreted cautiously, recognising that MRI abnormalities in SBO may lag behind biochemical improvement; however, persistent infection or progression could not be excluded on imaging alone. PET imaging was not performed, and no surgical debridement was undertaken during the admission.

Clinically, the facial palsy persisted. The patient became profoundly deconditioned, remained bed-bound, developed prolonged hypoactive delirium and acute kidney injury stage 3. After multidisciplinary discussion and consideration of prognosis and goals of care, escalation to intensive care was deemed inappropriate. He died in the hospital a short time later.

## Discussion

This case demonstrates several important issues in the recognition and management of skull base osteomyelitis in very frail older adults.

First, diagnostic delay is common in skull base osteomyelitis (1–3, 5, 6, 14). Initial assessment appropriately considered more prevalent causes of postural hypotension, including polypharmacy, diabetic autonomic neuropathy and cardiovascular disease. However, chronic ear symptoms were not initially linked to his recurrent falls and orthostatic symptoms. Only the dramatic episode of external auditory canal haemorrhage triggered a review by an otorhinolaryngology specialist and focused skull base imaging. Earlier specialist assessment of persistent otitis externa refractory to topical therapy might have allowed diagnosis before facial palsy and systemic deterioration developed.

Second, facial nerve palsy and chronic otitis externa should raise suspicion of SBO even when CT brain imaging is normal. Infection spreads from the external auditory canal through fissures and foramina in the temporal bone to involve the skull base and adjacent cranial nerves. Approximately one-third of patients with SBO develop facial nerve palsy. In this case, the palsy was initially investigated as a possible recurrent stroke, delaying recognition of the unifying skull base pathology (7).

Third, the association between SBO and severe orthostatic hypotension in this case should be interpreted cautiously. Carotid sinus region inflammation offers a biologically plausible mechanism because baroreceptor afferents from the carotid sinus travel via Hering’s nerve to central autonomic pathways, and adjacent skull base inflammation may disrupt this reflex arc (8). Nonetheless, this mechanism was not directly proven radiologically or physiologically here. Unlike the previously reported case of SBO-associated syncope, our patient’s hypotension did not improve with treatment, which raises the possibility of multifactorial orthostatic hypotension (4). Alternative contributors include diabetic autonomic neuropathy, prior cerebrovascular disease, advanced frailty and deconditioning, and previous exposure to vasoactive and psychoactive medication. Relevant differential diagnoses, therefore, include neurogenic orthostatic hypotension, medication-related hypotension, volume depletion and reflex syncope. Formal tilt-table testing was not undertaken; in the context of already documented bedside orthostatic hypotension, advanced frailty and evolving focal otological disease, it was unlikely to have altered immediate inpatient management (9).

Fourth, treatment of SBO is prolonged and complex. In the absence of high-quality randomised trials, current practice is guided mainly by observational studies and expert consensus, generally favouring prolonged culture-directed antipseudomonal therapy, careful glycaemic control, local ear care and serial clinical review (3, 5, 6, 10, 11). Surgical debridement is usually reserved for selected patients with abscess formation, necrotic tissue, sequestrum, fungal disease or failure of medical therapy rather than employed routinely in every case (3, 5, 6). Mixed follow-up imaging should also be interpreted cautiously: conventional MRI may lag behind clinical or biochemical response, and persistent or even apparently progressive signal abnormality does not always equate to uncontrolled infection. When clinical, biochemical and conventional imaging findings diverge, FDG-PET/CT may provide useful adjunctive assessment of disease activity, although it was not performed in this case (3, 12, 13).

Finally, this case highlights how repeated prescribing of topical steroids and antibiotics in primary care, without reassessment or escalation to an otorhinolaryngology specialist, may inadvertently mask progressive necrotising otitis externa. Structured pathways for timely referral in adults with persistent otitis externa, particularly those with diabetes or cranial neuropathies, may facilitate earlier diagnosis and earlier treatment initiation, which could help limit progression to SBO and its devastating complications (1, 3, 5).

## Conclusion

This case shows that skull base osteomyelitis should be considered in the differential diagnosis of refractory orthostatic hypotension and recurrent falls in older patients with diabetes, persistent otogenic symptoms and cranial neuropathy. In this patient, adjacent carotid canal/carotid sinus region inflammation may have contributed to autonomic dysfunction, but a multifactorial mechanism remained likely.

## Data Availability

The original contributions presented in the study are included in the article/supplementary material, further inquiries can be directed to the corresponding author.
